# Supplementary material for: Non-invasive suppression of essential tremor via phase-locked disruption of its temporal coherence
Source: Nat Commun. 2021 Jan 13;12:363. doi: 10.1038/s41467-020-20581-7 (PMC7806740; doi:10.1038/s41467-020-20581-7)
Supplement: Supplementary file 1 — Supplementary information [file 41467_2020_20581_MOESM1_ESM.pdf]

Supplementary Information for:

Non-invasive Suppression of Essential Tremor via Phase-Locked Disruption of its Temporal Coherence

1. Neurophysiological model

To explore the neurophysiological mechanism of our stimulation strategy we used a computational model of the Cortico-cerebello-thalamo-cortical (CCTC) network under essential tremor (ET) condition[1] and tested the effect of phase-locked stimulation of the cerebellum on the spiking dynamics. The model consisted of 425 single-compartment, conductance-based neurons from the olivocerebellar and thalamocortical loops, including 40 inferior olivary nucleus (ION) neurons in the brainstem, 200 Purkinje cells (PCs) and 20 granular layer clusters (GrL; 3 distinct neurons per cluster, 60 neurons altogether) in the cerebellar cortex, 5 glutamatergic deep cerebellar projection neurons (DCNs) and 5 nucleoolivary (NO) neurons in the dentate nucleus, 5 ventral intermediate thalamus (Vim) thalamocortical (TC) neurons, 100 pyramidal neurons (PYN), and 10 fast-spiking interneurons (FSI). Supplementary Fig. 5a shows a schematic representation of the model (see Methods for details, contextualized to hitherto approaches). As in our previous study[1], the power spectrum density (PSD) between 4 –12Hz of the ventral intermediate thalamus (Vim) was used as a proxy to the tremor activity. Supplementary Fig. 3b shows a representative PSD of the Vim under normal and ET conditions with a pathologic oscillation peak at 7Hz.

We first assessed the effect of periodic electrical stimulation of the cerebellum without phase-locking by adding sinusoidal input currents to the Purkinje cells (PCs) which were chosen as the stimulation target due to their abundance in the cerebellar cortex[2] and their high excitability (see Supplementary Fig. 4a-b for comparison with granular cells). We quantified the change in the spiking activity due to stimulation at the tremor frequency and a range of amplitudes evoking small sub-threshold depolarization as expected in our experiment (see Methods for details). We found that the stimulation entrained the PCs with an efficiency that increased with the current amplitude (Supplementary Fig. 3c). However, the entrainment of the PCs resulted in only a small decrease (<5%) or increase (<10%) in the tremor PSD of the Vim (Fig. Supplementary 3d, see also Supplementary Fig. 4c for representative spiking activity).

We then assessed the effect of phase-locking the stimulation to the tremor activity by computing the instantaneous phase of the TC neurons' spike-train online using ecHT and adjusting the phase of the sinusoidal currents to maintain a fix phase-lag as we did in our experiment (see Methods for details). We found that in this case, a narrow range of stimulating phase-lag values were capable of perturbing the synchronous activity of the PCs (Supplementary Fig. 3e) resulting in a large decrease (up to ~95%) in the tremor PSD of the Vim (Supplementary Fig. 3f). Higher current amplitudes resulted in a larger decrease in tremor-PSD and a wider range of efficacious phase-lags. Stimulating phase-lag values outside this range could result in a small increase (up to ~10%) in the tremor PSD of the Vim. Increasing the number of cells in the model affected the range of efficacious phase-lags but not the resulted drop in tremor PSD (Supplementary Fig. 4d).

At efficacious phase-lags, the hyperpolarizing phase of the stimulating currents was consistently aligned with the onset time of the complex spikes in the PCs, resulting in a gradual suppression of the periodic complex spiking and restoration of more physiological simple spiking (Supplementary Fig. 3g). The perturbation of the complex spiking in the PCs disrupted the periodicity of the spiking in the inferior olivary nucleus (ION) neurons (Supplementary Fig. 3h) and the temporal coherence of the bursting activity in the deep cerebellar neurons (DCNs; Supplementary Fig. 3i) thus ceasing the tremulous coherent drive to the Vim of the thalamocortical loop (Supplementary Fig. 3j). In contrast, at 180° relative phase-lags, the depolarizing phase of the stimulating currents was consistently aligned with the onset time of the complex spikes in the PCs, resulting in a small augmentation of the periodic complex spiking that led to a small reinforcement of the aberrant tremulous drive to the Vim of the thalamocortical loop (Supplementary Fig. 3g-j).

2. Supplementary figures

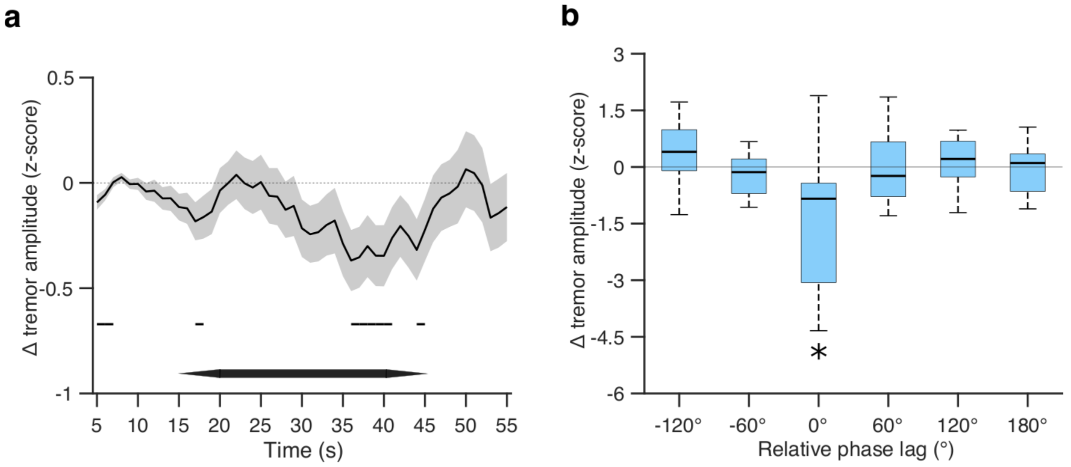

Supplementary Fig. 1 Tremor amplitude during phase-locked stimulation with values expressed relative to stimulation without phase-locking (related to **Fig. 3e** and **Fig. 4c**). **a** Change in tremor amplitude over time with stimulating currents were phase-locked to the tremor movement, showing the same as in **Fig. 3e**, but z-score values were expressed relative to non-phase-locked stimulation (instead of sham), by subtracting the median z-score during non-phase-locked stimulation. **b** Change in tremor amplitude versus stimulation phase lag, showing the same as in **Fig. 4c**, but z-score values were expressed relative to non-phase-locked stimulation (instead of sham), by subtracting the median z-score during non-phase-locked stimulation.

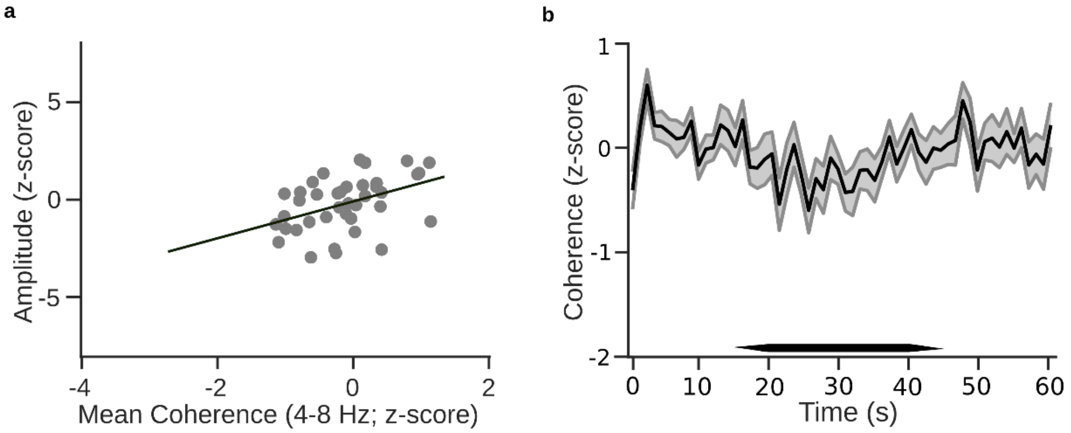

Supplementary Fig. 2 Temporal coherence analysis of trials during stimulation without phase-locking (related to Figure 6h). **a** Correlation between change in tremor's amplitude and change in tremor's temporal coherence at the tremor frequency band (i.e., 4-8 Hz) of trials with stimulation without phase-locking. Each datapoint corresponds to the z-score of tremor's amplitude versus the z-score tremor's temporal coherence of a single trial during the stimulation period relative to the baseline period. c=-0.008, m=0.95, R^2 =0.18 (c, line y-intercept; m, line slope). It shows that in the case of non-phase-locked stimulation, the change in the tremor amplitude was only weakly correlated (lower R^2) with the change in the tremor temporal coherence and exhibited a weaker dependency (smaller line slope, i.e., coefficient of gradient) compared to phase-locked stimulation (Fig. 6hii). **b** Change in tremor's temporal coherence at the tremor frequency band over time during stimulation without phase-locking. Shown values are mean ± st.d. z-score at each epoch during stimulation period relative to the mean and st.d. across epochs during baseline period for the non-phase locking trials.

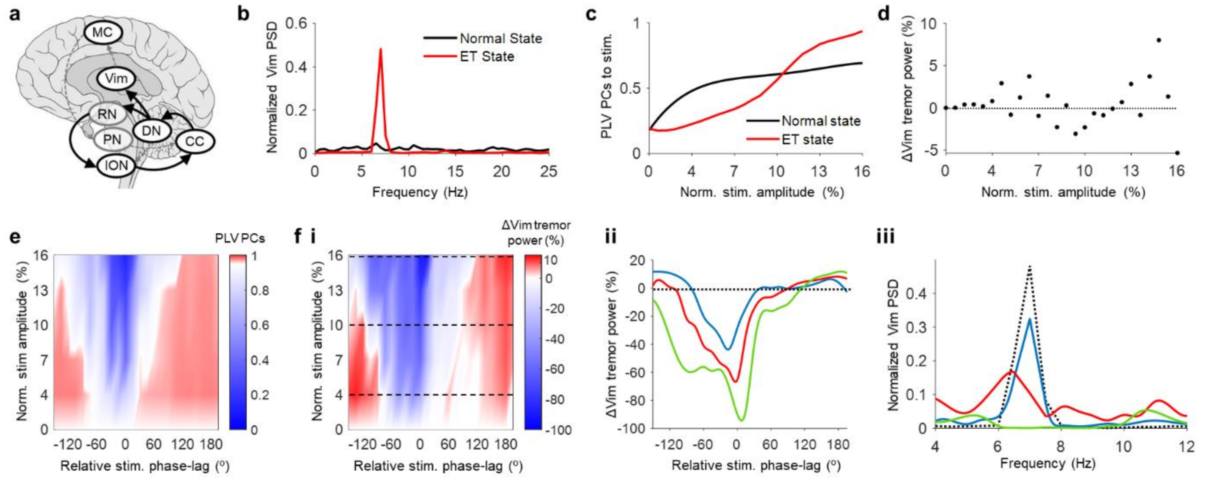

Supplementary Fig. 3 Neurophysiological modelling, narrow phasic perturbation of cerebellar PCs' synchrony can lead to large suppression of tremor amplitude. a Schematic of the Cortico-cerebello-thalamo-cortical (CCTC) network model; solid arrows and grey dashed arrows, pathways associated and not associated with ET generation, respectively; CC, cerebellar cortex; DN, dentate nucleus; RN, red nucleus; PN, pontine nucleus; Vim, ventral intermediate nucleus; MC, motor cortex. See Methods and Zhang et al.[1] for a detailed description of the model. **b** Normalized power spectral density (PSD) of the spike count trace of TC neurons in the Vim during tremor free condition, black line, and ET condition,

red line (tremor frequency 7Hz). ET condition is used throughout the rest of the figure. PSD values were normalized to total power between 0Hz and 25Hz. Simulation duration, 11.5s. **c-d** Sinusoidal current stimulation of PCs without phase-locking. Stimulation frequency, 7Hz. Current amplitude values were normalized to the amplitude of the endogenous synaptic current measured at rest; see Methods for details. **c** Modulation of PCs phase-locking value (PLV) versus normalized amplitude of stimulation during normal condition, black line, and ET condition, red line; see Methods for details of PLV computation. **d** Modulation of tremor PSD in Vim (i.e., maximal PSD between 4 -12Hz), showing percentage change in tremor PSD with respect to ET without stimulation. **e-k** Sinusoidal current stimulation of PCs phase-locked to Vim's tremor spike train; (see Methods for details of online phase-locking computation. Phase lag values are expressed relative to 330° phase lag showing the largest reduction in tremor amplitude and wrap to ±180°. **e** Modulation of PCs PLV, showing colormap of PLVs versus normalized amplitude and phase of stimulation. **f** Modulation of tremor PSD in Vim, shown are (i) colormap of percentage change in tremor PSD versus normalized amplitude and phase of stimulation; black dashed line, normalized stimulation amplitude of 4%, 10% and 16%, (ii) line plots of percentage change in tremor PSD in Vim versus stimulation phase for amplitudes 4% (blue line), 10% (red line), and 16% (green line), and (iii) Normalized Vim PSD during stimulation with a relative phase of 0° and the same normalized amplitudes.

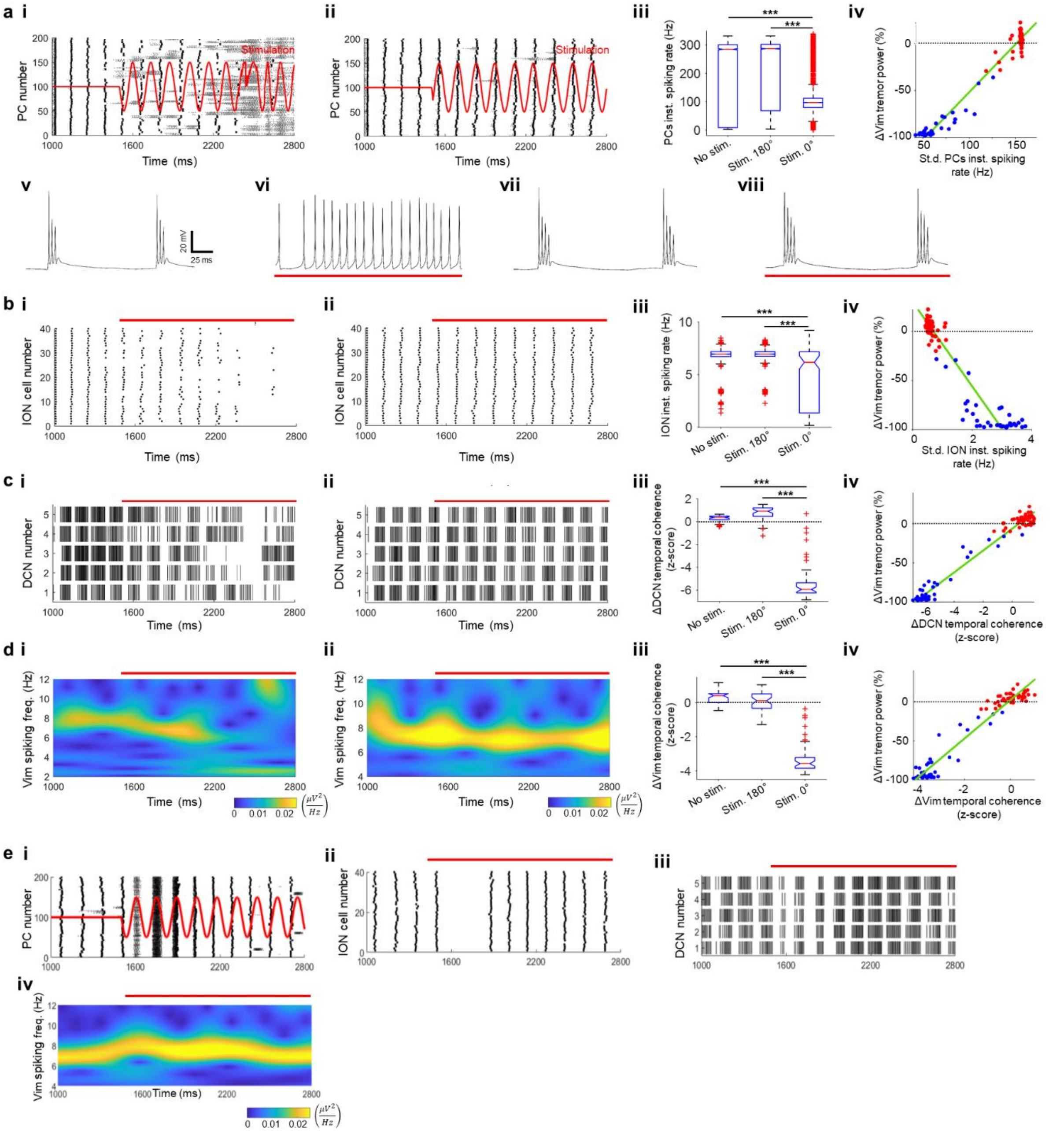

Supplementary Fig. 4 Neurophysiological modelling, efficacious phasic perturbation is mechanistically attributed to suppression of PCs' complex spikes. a Representative spiking activity of PCs, showing (i) spike raster plot during stimulation at 16% normalized amplitude and 0° relative phase-lag (black) and waveform of stimulating current (red); stimulation started after 1,500ms and lasted till the end of the simulation; (ii) same as (i) but at 180° relative phase-lag; (iii) statistics of PCs' instantaneous spiking rate (i.e., 1/inter-spike-interval) during no stimulation ('No stim.'), stimulation at 180° relative phase-lag ('Stim. 180°'), and stimulation at 0° relative phase-lag ('Stim. 0°'); red line, median; box edges, 25% and 75% percentiles; *** Stim-0 vs. No-stim, $p=4\cdot10^{-32}$; Stim-0 vs. Stim-180, $p=1.5\cdot10^{-12}$; Bartlett's test and post-hoc two-sample F-test with Bonferroni corrections for multiple comparisons; n=42 simulation repetitions with amplitudes equally sampled between 1% and 16%; (iv) correlation between change in Vim tremor PSD and standard deviation (st.d.) of PCs' instantaneous spike firing rate; c=-154, m=1.3, R^2 =0.97 (c, line y-intercept; m, line slope; green line, linear fit); stimulation at 0° relative phase-lag resulted in a breakdown of complex spikes (high median

instantaneous spiking rate due to short inter spikelet intervals, high st.d. instantaneous spiking rate due to long inter burst intervals) and restoration of regular simple spikes; (v-vi) representative spike train of a PC before (v) and during (vi) stimulation at 0° relative phase-lag; (vii-viii) same as (v-vi) but for 180° relative phase-lag. **b** same as (**a**) but showing results for ION neurons; red horizontal bar indicates stimulation period; (iii) *** $p=6{\cdot}10^{-9}$, $p=2.2{\cdot}10^{-12}$ (iv) c=30, m=-43, $R^2=0.9$. **c** same as (**b**) but showing results for DCNs, (iii) change in DCNs' spiking temporal coherence z-scored relative to mean and st.d. during baseline, *** $p=1.6{\cdot}10^{-31}$, $p=3.6{\cdot}10^{-26}$; (iv) correlation between change in Vim tremor PSD and change in DCNs' temporal coherence shown in (iii); c=-5.8, m=14, $R^2=0.97$. **d** same as (**c**) but showing results for TC neurons in Vim, with (i-ii) showing representative spiking spectrograms instead of spike raster plots, (iii) *** $p=7.7{\cdot}10^{-29}$, $p=2.0{\cdot}10^{-25}$; (iv) c=2.5, m=25, $R^2=0.95$. **e** Representative spiking activity during stimulation without phase-locking at the same normalized amplitude, (i-iv) showing the same as in (**ai-di**).

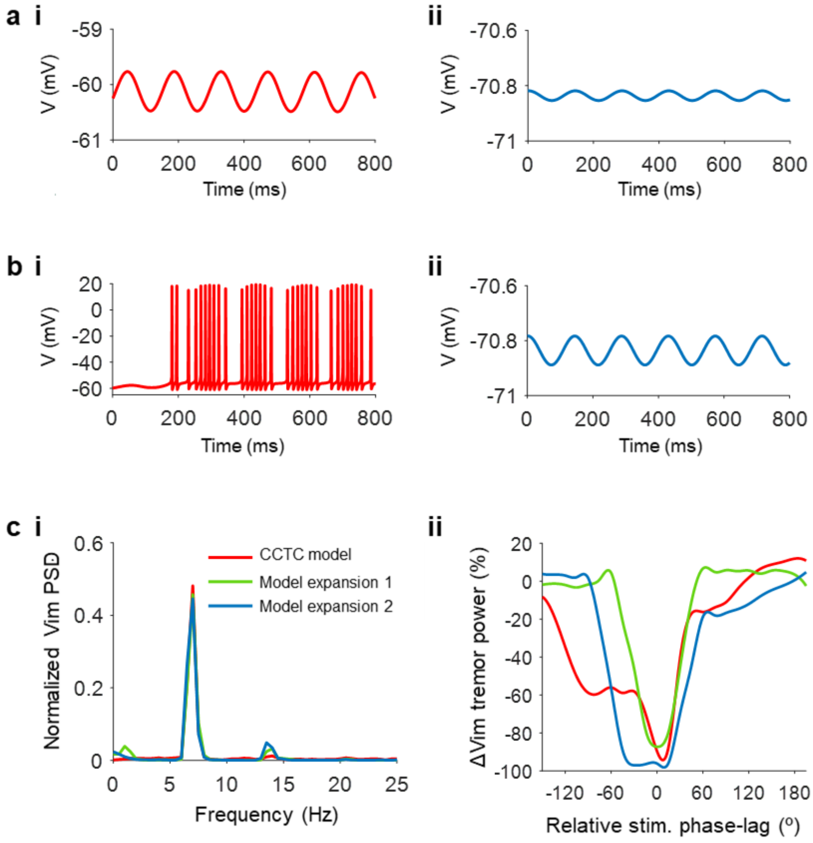

Supplementary Fig. 5 Neurophysiological modelling, sensitivity to cell type and model size. a-b, Direct effect of the stimulation on Purkinje cell (PC) and granule cell (GrC) in the cerebellar cortex. see Methods for details description of the PC and GrC models. **a** Representative traces of somatic transmembrane potential of (i) PC and (ii) GrC during stimulation with an external sinusoidal electric field oscillating at the tremor frequency (i.e., 7Hz) and having an amplitude of 2 V/m expected in our experiment (**Fig. 2b**). **b**, Same as (**a**) but for electric field having an amplitude of 6 V/m. The results shown in (a-b) indicate that the direct response of the cerebellar cortex to the stimulating electric fields is dominated by the PCs. **c** Effect of the model size on the simulation outcome. red line original 'CCTC model'; green line, 'Model expansion, 1'; blue line, 'Model expansion 2'; see Methods for details

description of the models. Showing (i) normalized Vim PSD during stimulation with a relative phase of 0° and normalized amplitude of 16% and (ii) percentage change in tremor PSD in Vim versus stimulation phase for the same stimulation. It shows that in comparison to the original model used in **Supplementary Fig. 3**, an increase in the number of cells in the model does not abolish the ET oscillation in the CCTC network (quantified via the PSD at the tremor frequencies in the Vim) and its response to phase-locked stimulation. However, it may modify range of efficacious phase-lags.

3. Supplementary tables

Supplementary Table 1 (related to Fig. 2-6)

Demographic and clinical information of participants. yrs, years; CRST, clinical rating scale for tremor. The demographic information in this table was published with the consent of the participants.

Participant No.	*Sex*	*Handed-ness*	*Stim Hemi sphere*	*Age (yrs)*	*Age at Onset (yrs)*	*Disease Duration (yrs)*	*Tremor Severity (CRST)*	*Stimulation Intensity (mA)*	*Tremor Frequency[1] (Hz)*	*Tremor Amplitude Baseline[1] (a.u.)*
1	m	r	l	65	5	60	48	2	5.2 (0.08, 0.9)	704 (0.2, 09)
2	f	l	l	48	45	3	14	4	6.9 (0.07, 1)	88 (0.2, 1)
3	m	r	r	80	55	25	16	2	6.2 (0.1, 0.9)	35 (0.2, 0.9)
4	f	r	l	79	69	10	29	2	5.9 (0.08, 0.9)	151 (0.1, 1)
5	m	r	r	71	25	46	44	1	4.6 (0.2, 0.6)	1613 (0.2, 02)
6	m	r	r	38	18	20	30	3	6.2 (0.09, 1)	276 (0.2, 0.2)
7	m	r	r	79	55	24	93	4	3.8 (0.09, 0.7)	1454 (0.2, 0.9)
8	f	r	r	78	57	21	57	3	5.4 (0.1, 0.9)	480 (0.2, 09)
9	m	l	r	52	1	51	57	2	6.2 (0.07, 0.9)	204 (0.1, 02)
10	m	r	l	82	64	18	63	3	5.2 (0.03, 0.9)	79 (0.1, 0.8)
11	m	r	l	70	55	15	37	3	4.1 (0.1, 1)	76 (0.2, 0.8)

[1] Showing in brackets participants' coefficient of variances (COVs; left) of tremor frequency and tremor amplitude during baseline period across trials and the corresponding p-values of the Hartigan's dip test for multimodality (right).

Demographic and clinical information statistics of all participant cohort ('all participants'), participants who responded to phase-locking stimulation ('responders'), and participants who did respond to phase-locking stimulation ('non-responders'), as well as significant difference (i.e., p-value) between responders and non-responders, characterized using Wilcoxon rank-sum test.

	all participants	responders	non-responders	p-value
Age (yrs)	67.5 ±15	61.6 ±16	77.8 ±4.7	0.07
Disease Duration (yrs)	26.6 ±17.9	27.8 ±20.3	24.5 ±15.4	0.78

Tremor Severity (CRST)	44.4 ±23	37 ±18	57.2 ±28	0.29
Stimulation Intensity (mA)	2.6 ±0.9	2.8 ±0.9	2.5 ±1.3	0.8
Tremor Frequency (Hz)	5.4 ±1.0	6 ±1.1	4.9 ±0.9	0.08
Tremor Amplitude Baseline (a.u.)	468 ±594	205 ±237	825 ±823	0.23

Demographic and clinical information during repetition experiment with a subset of participants

Participant No.	*Age (yrs)*	*Disease Duration (yrs)*	*Tremor Severity (CRST)*	*Stimulation Intensity (mA)*	*Tremor Frequency (Hz)*	*Tremor Amplitude Baseline (a.u.)*
1	68	63	75	2	4,9	974,0
2	51	6	15	3	6,5	43,0
3	84	29	47	2	6,4	53,0
6	42	24	31	3	6,8	82,0
9	55	54	47	2	6,9	118,0
11	73	18	46	3	4,8	46,0

Supplementary Table 2 (related to Fig. 2d)

Similarity of the mean phase lag between the stimulation conditions, assessed using Fisher test during the whole stimulation period (‘whole stim’), the 1st half of the stimulation period (‘1st stim half’), and the second half of the stimulation period (‘2nd stim half’). no-pl, no phase-locking.

Post-hoc analysis (p-value)

Set phase lags	Whole stim.	1st stim half	2nd stim half
0° vs 60°	2.73E-06	2.73E-06	2.73E-06
0° vs 120°	2.73E-06	2.73E-06	2.73E-06
0° vs 180°	0.20*	0.20*	0.20*
0° vs 240°	2.73E-06	2.73E-06	2.73E-06
0° vs 300°	2.73E-06	2.73E-06	2.73E-06
60° vs 120°	2.73E-06	2.73E-06	2.73E-06
60° vs 180°	2.73E-06	2.73E-06	2.73E-06
60° vs 240°	0.20	0.09	0.67
60° vs 300°	2.73E-06	2.73E-06	2.73E-06
120° vs 180°	2.73E-06	2.73E-06	2.73E-06
120° vs 240°	2.73E-06	2.73E-06	2.73E-06
120° vs 300°	0.39	0.67	0.01
180° vs 240°	2.73E-06	2.73E-06	2.73E-06
180° vs 300°	2.73E-06	2.73E-06	2.73E-06
240° vs 300°	2.73E-06	2.73E-06	2.73E-06
no-pl vs 0°	0.09	0.67	0.20

no-pl vs 60°	0.67	0.20	0.67
no-pl vs 120°	0.20	0.67	1.00
no-pl vs 180°	0.03	0.67	0.20
no-pl vs 240°	0.67	0.09	0.67
no-pl vs 300°	0.20	0.67	0.67

* We found that the Fisher test between 0° phase lag and 180° phase lag using MATLAB Circstats[3] function 'circ_cmtest' is inaccurate due to the phase wrapping of the function.

Supplementary Table 3 (related to Fig. 2e)

Similarity of the circular spread of the phase lags, quantified via the mean resultant vector length *R*, between the stimulation conditions, assessed using ANOVA with post-hoc analysis using Wilcoxon signed-rank test during the whole stimulation period ('whole stim'), the 1st half of the stimulation period ('1st stim half'), the second half ('2nd stim half') of the stimulation period, and between the 1st and 2nd halves of the stimulation period ('1st vs 2nd stim halves'). no-pl, no phase-locking.

Set phase lags	*Whole stim (p-value)*	*1st stim half (p-value)*	*2nd stim half (p-value)*
0° vs 60°	0.70	1.00	0.15
0° vs 120°	0.64	0.58	0.52
0° vs 180°	0.58	0.52	0.41
0° vs 240°	0.24	0.37	0.15
0° vs 300°	0.83	0.64	0.41
60° vs 120°	0.24	0.37	0.58
60° vs 180°	0.64	0.70	0.58
60° vs 240°	0.70	0.37	0.90
60° vs 300°	0.52	0.52	0.21
120° vs 180°	0.12	0.07	1.00
120° vs 240°	0.15	0.05	1.00
120° vs 300°	0.90	0.17	0.52
180° vs 240°	0.32	1.00	0.70
180° vs 300°	0.52	0.90	0.46
240° vs 300°	0.46	0.90	0.24
no-pl vs 0°	9.77E-04	9.77E-04	9.77E-04
no-pl vs 60°	9.77E-04	9.77E-04	9.77E-04
no-pl vs 120°	9.77E-04	9.77E-04	9.77E-04
no-pl vs 180°	9.77E-04	9.77E-04	9.77E-04
no-pl vs 240°	9.77E-04	9.77E-04	9.77E-04
no-pl vs 300°	9.77E-04	9.77E-04	9.77E-04
sham vs 0°	9.77E-04	9.77E-04	9.77E-04
sham vs 60°	9.77E-04	9.77E-04	9.77E-04
sham vs 120°	9.77E-04	9.77E-04	9.77E-04
sham vs 180°	9.77E-04	9.77E-04	9.77E-04
sham vs 240°	9.77E-04	9.77E-04	9.77E-04
sham vs 300°	9.77E-04	9.77E-04	9.77E-04

sham vs no-pl	0.37	0.27832	0.90

1st vs 2nd stim halves (p-value)

Set phase lag 1st stim half/2nd stim half	0°	60°	120°	180°	240°	300°	no-pl	sham
0°	0.58	0.15	0.83	0.76	0.15	0.64	9.77E-04	9.77E-04
60°	0.90	0.52	0.37	0.97	0.64	0.83	9.77E-04	9.77E-04
120°	0.90	0.02	0.15	0.15	0.04	0.46	9.77E-04	9.77E-04
180°	0.58	0.70	0.76	0.76	0.58	0.52	9.77E-04	9.77E-04
240°	0.21	0.97	0.32	0.41	0.97	0.28	9.77E-04	9.77E-04
300°	0.12	0.41	0.58	0.76	0.90	0.21	9.77E-04	9.77E-04
no-pl	9.77E-04	9.77E-04	9.77E-04	9.77E-04	9.77E-04	9.77E-04	0.90	0.83
sham	9.77E-04	9.77E-04	9.77E-04	9.77E-04	9.77E-04	9.77E-04	0.24	0.21

Supplementary Table 4 (related to Fig. 3b,f)

Number of participants showing a statistically significant change in tremor amplitude due to non phase-locked stimulation and phase-locked stimulation during the 1st half of the stimulation period (‘1st stim half’), the second half (‘2nd stim half’) of the stimulation period, and after the end of the stimulation (‘post stim’).

Number of participants showing a statistically significant change ’in tremor amplitude (Fisher exact test against number of participants with no change, ‘No’)

	Non phase-lock stimulation			Phase-lock stimulation		
	No	Reduction	Increase	No	Reduction	Increase
1st stim half	3	5 (p=0.66)	3 (p=1)	1	5 (p=0.15)	5 (p=0.15)
2nd stim half	5	4 (p=0.66)	2 (p=1)	1	9 (p<0.005)	4 (p=0.31)
Post stim	3	5 (p=0.66)	3 (p=1)	0	10 (p<0.00005)	3 (p=0.21)

In the 2nd stim half period and post-stim period, 3 and 2 participants, respectively, showed a significant reduction in one phase-lag and a significant increase in another phase-lag. In addition, in the 2nd stim half period and post-stim period, 3 and 2 participants, respectively, showed a significant reduction during both stimulation with phase-locking and without phase-locking (the reduction during stimulation without phase-locking was however typically smaller).

Supplementary Table 5 (related to Fig. 4a,c)

Change in tremor amplitude induced by phase-locked stimulation during the 1st half of the stimulation period (‘1st stim half’), the second half (‘2nd stim half’) of the stimulation period, and after the end of the stimulation (‘post stim’).

Change in tremor amplitude vs stim phase lag (mean ±st.d. z-score, unpaired t-test p-value)

Set phase lag	1st stim half	2nd stim half	post stim
0°	-0.61 ± 0.38, p=0.14	-0.86 ± 0.39, p=0.05	-0.67 ± 0.37, p=0.10
60°	0.09 ± 0.26, p=0.75	-0.14 ± 0.26, p=0.60	-0.06 ± 0.25, p=0.80

120°	-0.27 ± 0.39, p=0.51	-0.54 ± 0.50, p=0.30	-0.71 ± 0.35, p=0.07
180°	-0.26 ± 0.20, p=0.23	-0.24 ± 0.37, p=0.53	-0.68 ± 0.30, p=0.05
240°	-0.04 ± 0.24, p=0.87	-0.08 ± 0.30, p=0.79	-0.49 ± 0.42, p=0.27
300°	-0.39 ± 0.45, p=0.41	-0.38 ± 0.39, p=0.35	-0.36 ± 0.33, p=0.29

Tremor amplitude vs stim phase lag expressed relative to phase of maximal reduction (mean ±st.d. z-score, unpaired t-test p-value)

Relative phase lag	1st stim half	2nd stim half*	Post stim
-120°	-0.15 ± 0.08, p=0.56	0.18 ± 0.07, p=0.43	-0.26 ± 0.08, p=0.37
-60°	0.18 ± 0.08, p=0.52	-0.22 ± 0.07, p=0.33	-0.27 ± 0.08, p=0.32
0°	-1.28 ± 0.13, p=0.02	-1.43 ± 0.12, p=0.01	-1.74 ± 0.10, p<0.0005
60°	-0.03 ± 0.07, p=0.89	-0.11 ± 0.09, p=0.72	-0.10 ± 0.07, p=0.67
120°	-0.39 ± 0.11, p=0.31	-0.57 ± 0.17, p=0.35	-0.23 ± 0.12, p=0.59
180°	0.19 ± 0.07, p=0.40	-0.10 ± 0.06, p=0.64	-0.37 ± 0.08, p=0.20

* Significance during the 2nd stim half was also tested using 2-sample Kolmogorov-Smirnov test against a surrogate distribution of z-score values yielding -120°, p=0.05; -60°, p=0.1; 0°, p=0.002; 60°, p=0.09; 120°, p=0.09; 180°, p=0.1.

Participants showing a statistically significant change in tremor amplitude due to phase-locked stimulation in the 2nd stim half. Showing participant number (‘ID’) and p-value of statistical testing comparing the phase-locked stimulation condition with sham stimulation (‘p-value Shm’). For subjects who also showed a significant reduction in tremor amplitude during non-phase-locked stimulation, the p-value of statistical testing comparing the phase-locked stimulation condition with the non-phase-locked stimulation condition (‘p-value non-pl’) is added.

Participants showing a statistically significant change in tremor amplitude vs stim phase lag (ID, p-value Shm, p-value non-pl)

Set phase lag	Tremor decrease	Tremor increase
0°	1 ($3\cdot10^{-4}$), 2 ($3\cdot10^{-3}$, 0.2), 3 ($2\cdot10^{-7}$), 4 ($6\cdot10^{-3}$), 6 ($1\cdot10^{-5}$)	5 ($7\cdot10^{-3}$, 0.5)
60°	4 ($3\cdot10^{-4}$), 6 ($1\cdot10^{-3}$), 8 ($5\cdot10^{-4}$, $1\cdot10^{-3}$), 9 ($6\cdot10^{-3}$), 10 ($2\cdot10^{-4}$, 0.9)	5 ($8\cdot10^{-4}$, 0.3)
120°	2 ($4\cdot10^{-4}$, 0.06), 6 ($1\cdot10^{-3}$), 7 ($4\cdot10^{-5}$)	4 ($7\cdot10^{-5}$), 5 ($2\cdot10^{-3}$, $1\cdot10^{-3}$)
180°	2 ($3\cdot10^{-8}$, $1\cdot10^{-5}$), 8 ($1\cdot10^{-4}$, $2\cdot10^{-4}$)	4 ($4\cdot10^{-3}$), 5 ($1\cdot10^{-5}$, 0.04)
240°	2 ($4\cdot10^{-4}$, $8\cdot10^{-3}$), 8 ($3\cdot10^{-3}$, 0.08), 10 ($2\cdot10^{-5}$, 0.5)	5 ($1\cdot10^{-7}$, $4\cdot10^{-3}$), 7 ($3\cdot10^{-4}$, 0.2)
300°	2 ($3\cdot10^{-7}$, $3\cdot10^{-7}$)	10 ($2\cdot10^{-3}$)

Number of participants showing a statistically significant change in tremor amplitude due to phase-locked stimulation during 2nd stim half, computed using Fisher exact test against the number of participants who did not show any change in tremor amplitude.

Number of participants showing a statistically significant change in tremor amplitude vs stim phase lag

Set phase lag	Tremor decrease	Tremor increase

	(n decrease, n no-change, p-value)	(n increase, n no-change, p-value)
0°	5, 5, p=1	1, 5, p=0.15
60°	5, 5, p=1	1, 5, p=0.15
120°	3, 6, p=0.39	2, 6, p=0.18
180°	2, 7, p=0.08	2, 7, p=0.08
240°	3, 6, p=0.39	2, 6, p=0.18
300°	1, 9, p<0.005	1, 9, p<0.005

Number of participants showing a statistically significant change in tremor amplitude vs stim phase lag expressed relative to phase of maximal reduction

Set phase lag	Tremor decrease (n decrease, n no-change, p-value)	Tremor increase (n increase, n no-change, p-value)
-120°	1, 9, p<0.005	1, 9, p<0.005
-60°	2, 7, p=0.08	2, 7, p=0.08
0°	9, 1, p<0.005	1, 1, p=1
60°	3, 6, p=0.39	2, 6, p=0.18
120°	2, 8, p=0.03	1, 8, p=0.01
180°	2, 7, p=0.08	2, 7, p=0.08

Supplementary Table 6 (related to Fig. 4e-h)

Similarity of the mean phase-lag between the original experiment and the repeated experiment (n=6, including participants 1,2,3,6, 9, and 11), assessed using Circular Krusical Wallis test.

Phase-lag (mean ±st.d., p-value)

Set phase lag	original experiment	repeated experiment	difference
0°	357º ± 13º	2º ± 6º	p=0.76
60°	60º ± 11º	60º ± 7º	p=0.77
120°	121º ± 9º	110º ± 8º	p=0.19
180°	182º ± 16º	178º ± 6º	p=0.75
240°	242º ± 3º	237º ± 8º	p=0.37
300°	299º ± 12º	302º ± 9º	p=0.53

Similarity of the phase resultant between the original experiment and the repeated experiment (n=6, including participants 1,2,3,6, 9, and 11), assessed using a paired sign-rank test.

Phase resultant (mean ±st.d., p-value)

Set phase lag	original experiment	repeated experiment	difference
0°	0.98 ± 0.01	0.95 ± 0.04	p=0.09
60°	0.98 ± 0.01	0.96 ± 0.03	p=0.79
120°	0.98 ± 0.01	0.96 ± 0.04	p=0.84
180°	0.98 ± 0.01	0.96 ± 0.04	p=0.07
240°	0.98 ± 0.01	0.93 ± 0.06	p=0.08

300°	0.97 ± 0.02	0.95 ± 0.07	p=0.39

Supplementary Table 7 (related to Fig. 4g)

Similarity of the change in tremor amplitude induced by phase-locked stimulation between the original experiment and the repeated experiment (n=6, including participants 1,2,3,6, 9, and 11), assessed using paired t-test. Showing, results during the 1st half of the stimulation period ('1st stim half'), the second half ('2nd stim half') of the stimulation period, and after the end of the stimulation ('post stim').

Change in tremor amplitude (mean ±st.d. z-score, unpaired t-test p-value)

period	original experiment	repeated experiment	difference
1st stim half	-1.86 ± 0.39, p=0.0001	-1.82 ± 0.42, p=0.01	p=0.93
2nd stim half	-2.10 ± 0.46, p=0.001	-2.28 ± 0.53, p=0.001	p=0.83
Post stim	-1.81 ± 0.27, p=0.0001	-1.62 ± 0.27, p<0.0001	p=0.58

Participants showing a statistically significant change in tremor amplitude due to phase-locked stimulation between the original experiment and the repeated experiment (n=6, including participants 1,2,3,6, 9, and 11). Showing, results during the 1st half of the stimulation period ('1st stim half'), the second half ('2nd stim half') of the stimulation period, and after the end of the stimulation ('post stim').

Number of participants showing a statistically significant change in tremor amplitude vs stim phase lag

Set phase lag	Tremor decrease (n original experiment, n repeated experiment)	Tremor increase (n original experiment, n repeated experiment)
1st stim half	4, 4	1, 2
2nd stim half	5, 5	0, 3
Post stim	6, 5	0, 2

Supplementary Table 8 (related to Fig. 4h)

Similarity of the change in tremor amplitude during stimulation between the original experiment and the repeated experiment (n=6, including participants 1,2,3,6, 9, and 11), assessed using paired t-test.

Change in tremor amplitude vs stim phase lag (mean ±st.d. z-score)

Set phase lag	original experiment	repeated experiment	difference (p-value)
0°	-1.46 ± 0.59	-0.33 ± 0.56	p<0.00001
60°	-0.20 ± 0.23	-0.28 ± 0.46	p=0.33
120°	-0.92 ± 0.80	-0.94 ± 0.44	p=0.10
180°	-0.32 ± 0.59	-0.98 ± 0.70	p=0.37
240°	-0.08 ± 0.31	-0.88 ± 0.44	p=0.10
300°	-0.91 ± 0.61	-0.92 ± 1.19	p=0.03

Supplementary Table 9 (related to Fig. 5c-d)

Most informative features, i.e., the features shown in **Fig. 5c** at the centres of the clusters of correlated features, found to predict the participants' response to stimulation. See Fulcher et al.[4] for description of the features.

Feature ID in [4]	Feature name	Classification accuracy (%)
7588	MF_GARCHfit_ar_P1_Q1_diff_ac1	79.22
1129	CO_trev_3_raw	78.98
916	EN_PermEn_2_1_normPermEn	80.75
1125	CO_trev_2_abs	77.76
7467	MF_armax_2_2_05_1_maxdc	83.10
4138	SC_FluctAnal_2_dfa_50_3_logi_r1_se1	83.61
4141	SC_FluctAnal_2_dfa_50_3_logi_r1_resac1	79.32
4568	SP_Summaries_fft_linfitloglog_all_a2	78.69
5185	NL_BoxCorrDim_50_ac_5_mind2	78.03
16	rms	78.44
3561	SB_MotifTwo_mean_uuuu	78.70
2390	FC_Surprise_T1_50_3_udq_500_std	80.41
1900	CO_StickAngles_y_q1_all	77.98
3289	DN_OutlierInclude_n_001_nfla	77.53

Description of features shown in Fig. 5d

- Normalised non-linear autocorrelation trev function (feature ID, 1129) – The function calculates the mean of the difference between the shifted time-series. A perfectly sinusoidal timeseries should have a zero difference in the means when shifted by tau. In our data, the non-responders had a more negative trev value which suggest that their movement was less symmetric.
- Root-mean square (feature ID, 16) – The function computed the root mean square of the time series (i.e., amplitude). In our data, the responders had lower RMS.
- Motif correlation (feature ID, 3561) – The function searches for local motifs in a binary symbolisation of the time series with data points larger than mean set to 1 and those below the mean to 0. In our data, non-responders had larger amplitude above the mean, i.e., less symmetry relative to the time axis.
- Predictive memory of signal (feature ID 2390) – The function estimates the surprise in the next data point given recent memory of the previous data points and then computes the st.d. of the surprise matrix. In our data, non-responders had a higher portion of extreme values compared to the mean information, thus more information in each data point.

See **Supplementary Software** for MATLAB code computing the abovementioned features.

Supplementary Table 10 (related to Fig. 5g)

Demographic and clinical information of second cohort of participants. yrs, years; CRST, clinical rating scale for tremor; a.u., arbitrary units. The demographic information in this table was published with the consent of the participants.

Partici-pant No.	*Sex*	*Handed-ness*	*Stimulated Hemisphere*	*Age (yrs)*	*Age at Onset (yrs)*	*Disease Duration (yrs)*	*Tremor Severity (CRST)*	*Stimulation Intensity (mA)*	*Tremor Frequency (Hz)*	*Tremor Amplitude Baseline (a.u.)*
1	m	r	r	61	20	41	14	2	8.1	44.0
2	f	r	r	43	37	6	7	3	8.7	20.0
3	m	r	r	56	26	30	25	1	7.4	37.0
4	m	l	l	47	15	32	31	3	5.4	71.0
5	f	r	r	54	44	10	7	2	6.8	17.0
6	f	r	l	42	38	4	6	1	8.5	16.0
7	f	r	l	80	42	38	65	1	4.2	220.3

Demographic and clinical information statistics from the second cohort of participants, showing all participant cohort ('all participants'), participants who responded to phase-locking stimulation ('responders'), and participants who did respond to phase-locking stimulation ('non-responders'), as well as significant difference (i.e., p-value) between responders and non-responders, characterized using Wilcoxon rank-sum test.

	all participants	responders	non-responders	p-value
Age (yrs)	55 ± 13	60 ± 19	51 ± 8	0.63
Disease Duration (yrs)	23 ± 16	25 ± 17	22 ± 18	1.0
Tremor Severity (CRST)	22 ± 21	32 ± 30	14 ± 12	0.46
Stimulation Intensity (mA)	0.2 ± 0.1	0.2 ± 0.1	0.2 ± 0.1	0.74
Tremor Frequency (Hz)	7.0 ± 1.7	6.8 ± 2.3	7.2 ± 1.4	1.0
Tremor Amplitude Baseline (a.u.)	60.8 ± 73.0	92.4 ± 111.1	37 ± 26.1	0.63

Supplementary Table 11 (related to Fig. 5g)

Phase-lag between stimulation and tremor movement in the second cohort of participants during the 1st half of the stimulation period ('1st stim half'), the second half ('2nd stim half') of the stimulation period, and after the end of the stimulation ('post stim').

Phase-lag (mean ±st.d., p-value)		
Set phase lag	1st stim half	2nd stim half
0°	7° ± 10°, p<0.00001	6° ± 12°, p<0.00001
60°	59° ± 9°, p<0.00001	60° ± 8°, p<0.00001
120°	113° ± 20°, p<0.00001	122° ± 14°, p<0.00001
180°	180° ± 11°, p<0.00001	183° ± 12°, p<0.00001
240°	247° ± 7°, p<0.00001	245° ± 9°, p<0.00001
300°	308° ± 7°, p<0.00001	302° ± 7°, p<0.00001

Circular spread of the phase lags quantified via the mean resultant vector length *R* in the second cohort of participants.

Phase resultant (mean ±st.d., p-value)		
Set phase lag	1st stim half	2nd stim half
0°	0.97 ± 0.03	0.96 ± 0.04
60°	0.96 ± 0.03	0.97 ± 0.02
120°	0.93 ± 0.14	0.94 ± 0.10
180°	0.96 ± 0.05	0.97 ± 0.02
240°	0.95 ± 0.07	0.97 ± 0.04
300°	0.94 ± 0.08	0.95 ± 0.06

Supplementary Table 12 (related to Fig. 5g)

participants in the second cohort, showing a statistically significant change (decrease or increase) in tremor amplitude during phase-locked stimulation and non phase-locked stimulation.

Number of participants showing a statistically significant change in tremor amplitude vs stim phase lag		
Set phase lag	Phase-locked stimulation (n decrease, n increase)	Non phase-locked stimulation (n decrease, n increase)
1st stim half	5, 2	3, 1
2nd stim half	4, 4	1, 2
Post stim	5, 4	1, 2

Change in tremor amplitude induced by phase- vs phase of stimulation in the second cohort of participants.

Change in tremor amplitude vs stim phase lag (mean ±st.d. z-score, unpaired t-test p-value)			
Set phase lag	1st stim half	2nd stim half	post stim
0°	-0.12 ± 0.86, p=0.72	-0.30 ± 2.31, p=0.74	-0.40 ± 2.92, p=0.73
60°	-0.03 ± 0.86, p=0.94	0.24 ± 1.69, p=0.72	-0.16 ± 1.79, p=0.82
120°	-0.19 ± 1.20, p=0.69	-0.18 ± 1.20, p=0.70	0.06 ± 1.38, p=0.91
180°	-0.36 ± 0.93, p=0.35	0.02 ± 1.39, p=0.97	-0.04 ± 1.52, p=0.95
240°	-0.37 ± 0.76, p=0.25	-0.17 ± 1.07, p=0.70	-0.36 ± 0.68, p=0.21
300°	-0.11 ± 0.73, p=0.70	0.15 ± 1.05, p=0.71	0.02 ± 1.24, p=0.96

Supplementary Table 13 (related to Fig. 6e-f)

Most informative features, i.e., the features shown in **Fig. 6e** at the centres of the clusters of correlated features, found to predict the participants' response to stimulation. See Fulcher et al.[4] for description of the features.

Feature ID in [4]	Feature name	Classification accuracy (%)
2315	FC_Surprise_T2_50_3_q_500_median	79.31
4432	SP_Summaries_welch_rect_fpoly2_r2	78.45

3077	FC_LocalSimple_median5_taures	75.86
526	CO_glscf_2_5_tau	75.86
6641	WL_DetailCoeffs_db3_max_max_median	77.59
1529	CO_Embed2_Basic_1_downdiag05	76.72
1927	CO_Embed2_tau_mean_eucdm	75.00
3286	DN_OutlierInclude_n_001_nfexpb	75.00
1938	CO_Embed2_tau_areas_50	75.00

Description of features shown in **Fig. 6f**

- Information Gain – The function estimates the predictability of the next data point given the previous data points. A perfect sine wave has zero median information gain.
  In our data, stimulation that suppressed the tremor amplitude increased the information gain.
- Quadratic fit of power spectrum cumulative sum - The function computes the R^2 of a quadratic fit to the cumulative sum of the power spectrum.
  In our data, stimulation that suppressed the tremor amplitude increase the accuracy of the fit, potentially by reducing the peak at the tremor frequency.

See **Supplementary Software** for MATLAB code computing the abovementioned features.

Supplementary References

1. Zhang, X. & Santaniello, S. Role of cerebellar GABAergic dysfunctions in the origins of essential tremor. *Proc. Natl. Acad. Sci. U. S. A.* (2019). doi:10.1073/pnas.1817689116

2. Deuschl, G., Raethjen, J., Hellriegel, H. & Elble, R. Treatment of patients with essential tremor. *The Lancet Neurology* (2011). doi:10.1016/S1474-4422(10)70322-7

3. Berens, P. CircStat : A MATLAB Toolbox for Circular Statistics . *J. Stat. Softw.* (2009). doi:10.18637/jss.v031.i10

4. Fulcher, B. D. & Jones, N. S. hctsa: A Computational Framework for Automated Time-Series Phenotyping Using Massive Feature Extraction. *Cell Syst.* (2017). doi:10.1016/j.cels.2017.10.001
