# Supplementary material for: Non-invasive suppression of essential tremor via phase-locked disruption of its temporal coherence
Source: Nat Commun. 2021 Jan 13;12:363. doi: 10.1038/s41467-020-20581-7 (PMC7806740; doi:10.1038/s41467-020-20581-7)
Supplement: Supplementary file 6 — Reporting Summary [file 41467_2020_20581_MOESM6_ESM.pdf]

nature research

Corresponding author(s): Nir Grossman

Last updated by author(s): Apr 6, 2020

Reporting Summary

Nature Research wishes to improve the reproducibility of the work that we publish. This form provides structure for consistency and transparency in reporting. For further information on Nature Research policies, see Authors & Referees and the Editorial Policy Checklist.

Statistics

For all statistical analyses, confirm that the following items are present in the figure legend, table legend, main text, or Methods section.

n/a	Confirmed	
☐	☒	The exact sample size (*n*) for each experimental group/condition, given as a discrete number and unit of measurement
☐	☒	A statement on whether measurements were taken from distinct samples or whether the same sample was measured repeatedly
☐	☒	The statistical test(s) used AND whether they are one- or two-sided<br>*Only common tests should be described solely by name; describe more complex techniques in the Methods section.*
☐	☒	A description of all covariates tested
☐	☒	A description of any assumptions or corrections, such as tests of normality and adjustment for multiple comparisons
☐	☒	A full description of the statistical parameters including central tendency (e.g. means) or other basic estimates (e.g. regression coefficient) AND variation (e.g. standard deviation) or associated estimates of uncertainty (e.g. confidence intervals)
☐	☒	For null hypothesis testing, the test statistic (e.g. *F*, *t*, *r*) with confidence intervals, effect sizes, degrees of freedom and *P* value noted<br>*Give P values as exact values whenever suitable.*
☒	☐	For Bayesian analysis, information on the choice of priors and Markov chain Monte Carlo settings
☐	☒	For hierarchical and complex designs, identification of the appropriate level for tests and full reporting of outcomes
☐	☒	Estimates of effect sizes (e.g. Cohen's *d*, Pearson's *r*), indicating how they were calculated

Our web collection on statistics for biologists contains articles on many of the points above.

Software and code

Policy information about availability of computer code

Data collection	Arduino ver 1.8.5 with custom made scripts, Processing ver 13, MATLAB (MathWorks Inc) ver 2017 with custom made scripts
Data analysis	MATLAB (MathWorks Inc) ver 2017 with custom made scripts

For manuscripts utilizing custom algorithms or software that are central to the research but not yet described in published literature, software must be made available to editors/reviewers. We strongly encourage code deposition in a community repository (e.g. GitHub). See the Nature Research guidelines for submitting code & software for further information.

Data

Policy information about availability of data

All manuscripts must include a data availability statement. This statement should provide the following information, where applicable:

- Accession codes, unique identifiers, or web links for publicly available datasets
- A list of figures that have associated raw data
- A description of any restrictions on data availability

Data Availability - The tremor data that support the findings of this study is available from the corresponding author upon request.
Code availability- Matlab codes for computing ecHT and most informative features in Figure 4 and Figure 5 are available as a Supplementary Software. The code for analysis of tremor measurements is available from the corresponding author upon request.

Field-specific reporting

Please select the one below that is the best fit for your research. If you are not sure, read the appropriate sections before making your selection.

☒ Life sciences ☐ Behavioural & social sciences ☐ Ecological, evolutionary & environmental sciences

For a reference copy of the document with all sections, see nature.com/documents/nr-reporting-summary-flat.pdf

Life sciences study design

All studies must disclose on these points even when the disclosure is negative.

Sample size	Sample size was estimated based on prior studies with tremor patients and brain stimulation.
Data exclusions	No data was excluded from the analysis.
Replication	Each stimulation condition was tested 4 times in a random order. Classification was performed using a 10-fold cross validation to reduce bias and variance.
Randomization	The study had a cross-over design, i.e., each participant was tested with all 8 stimulation conditions, 4 times in a random order.
Blinding	Both the investigator and the participants were blinded to the order of stimulation. Blindness of the investigator was achieved via a script based control of the stimulator.

Reporting for specific materials, systems and methods

We require information from authors about some types of materials, experimental systems and methods used in many studies. Here, indicate whether each material, system or method listed is relevant to your study. If you are not sure if a list item applies to your research, read the appropriate section before selecting a response.

Materials & experimental systems

n/a	Involved in the study	
☒	☐	Antibodies
☒	☐	Eukaryotic cell lines
☒	☐	Palaeontology
☒	☐	Animals and other organisms
☐	☒	Human research participants
☒	☐	Clinical data

Methods

n/a	Involved in the study	
☒	☐	ChIP-seq
☒	☐	Flow cytometry
☒	☐	MRI-based neuroimaging

Human research participants

Policy information about studies involving human research participants

Population characteristics	The ET patient cohort had the following demographic characteristics: age 67.5 ±15 years (mean ±st.d.), age of ET onset 40.8 ±24.2 years, disease duration 26.6 ±17.9 years and CRST 44.4 ±23.
Recruitment	Eleven ET patients (3 females) were recruited from the outpatient department of the UK National Hospital of Neurology and Neurosurgery, London. All patients fulfilled the diagnostic criteria for ET according to the consensus statement of the Movement Disorder Society 25 and were on a stable treatment regime for their tremor for at least 30 days prior to the experiment.
Ethics oversight	The study was approved by the research ethics committee of UCLH in accordance with the declaration of Helsinki. All participants provided written informed consent prior to study participation.

Note that full information on the approval of the study protocol must also be provided in the manuscript.
